# Supplementary material for: Stacking Model‐Based Classifiers for Dealing With Multiple Sets of Noisy Labels
Source: Biom J. 2025 Mar 12;67(2):e70042. doi: 10.1002/bimj.70042 (PMC11898607; doi:10.1002/bimj.70042)
Supplement: Supplementary file 1 — Supporting Information [file BIMJ-67-e70042-s001.zip › density-based-ensemble-model-main/competitors_real_data/netlab-master/netlab-master/help/demgmm1.htm]

Netlab Reference Manual demgmm1


# demgmm1

## Purpose

Demonstrate EM for Gaussian mixtures.

## Synopsis

```
demgmm1
```

## Description

This script demonstrates the use of the EM algorithm to fit a mixture
of Gaussians to a set of data using maximum likelihood. A colour
coding scheme is used to illustrate the evaluation of the posterior
probabilities in the E-step of the EM algorithm.

## See Also

`demgmm2`, `demgmm3`, `demgmm4`, `gmm`, `gmmem`, `gmmpost`

---

**Pages:**
Index


---

Copyright (c) Ian T Nabney (1996-9)
